# Scrotal calcinosis: two case reports

**DOI:** 10.1186/s13256-017-1451-8

**Published:** 2017-11-05

**Authors:** O. Karray, A. Dhaoui, R. Boulma, K. Bellil, H. Khouni

**Affiliations:** 1 Urology Unit, Inrerior security forces hospital, La Marsa, Tunisia; 2 Pathology Department, Inrerior security forces hospital, La Marsa, Tunisia

**Keywords:** Scrotum, Calcinosis, Surgery

## Abstract

**Background:**

Scrotal calcinosis is a rare and benign condition. It usually gives rise to few symptoms, and the impact is mainly functional and aesthetic. It is considered part of dystrophic calcinosis cutis. Surgical management is the only curative approach, and recurrence has been described in few cases.

**Case presentation:**

We report cases of two North African white patients with operated scrotal calcinosis. We describe the clinical and histological aspects as well as a pathogenic hypothesis and surgical management principles.

**Conclusions:**

A surgical approach to scrotal calcinosis must consider the aesthetic and functional aspects postoperatively. A complete excision prevents recurrence. Psychological support is required in association with surgery because the lesions are benign and concern an intimate part of the body.

## Background

Scrotal calcinosis is described as a skin condition of multiple hard, painless, asymptomatic cutaneous nodules without abnormalities in the phosphocalcic metabolism. It usually occurs in young adults with no histology of traumatism, genitourinary infections, or hormonal disorders. Diagnostic certitude is based on histology of the surgical resection specimen. Surgical resection should be as complete as possible, involving small nodular lesions, in order to rule out recurrence. The prognosis is favorable because, to our knowledge, malignant degeneration has not been described to date.

## Case presentations

### Patient 1

**Fig. 1:**
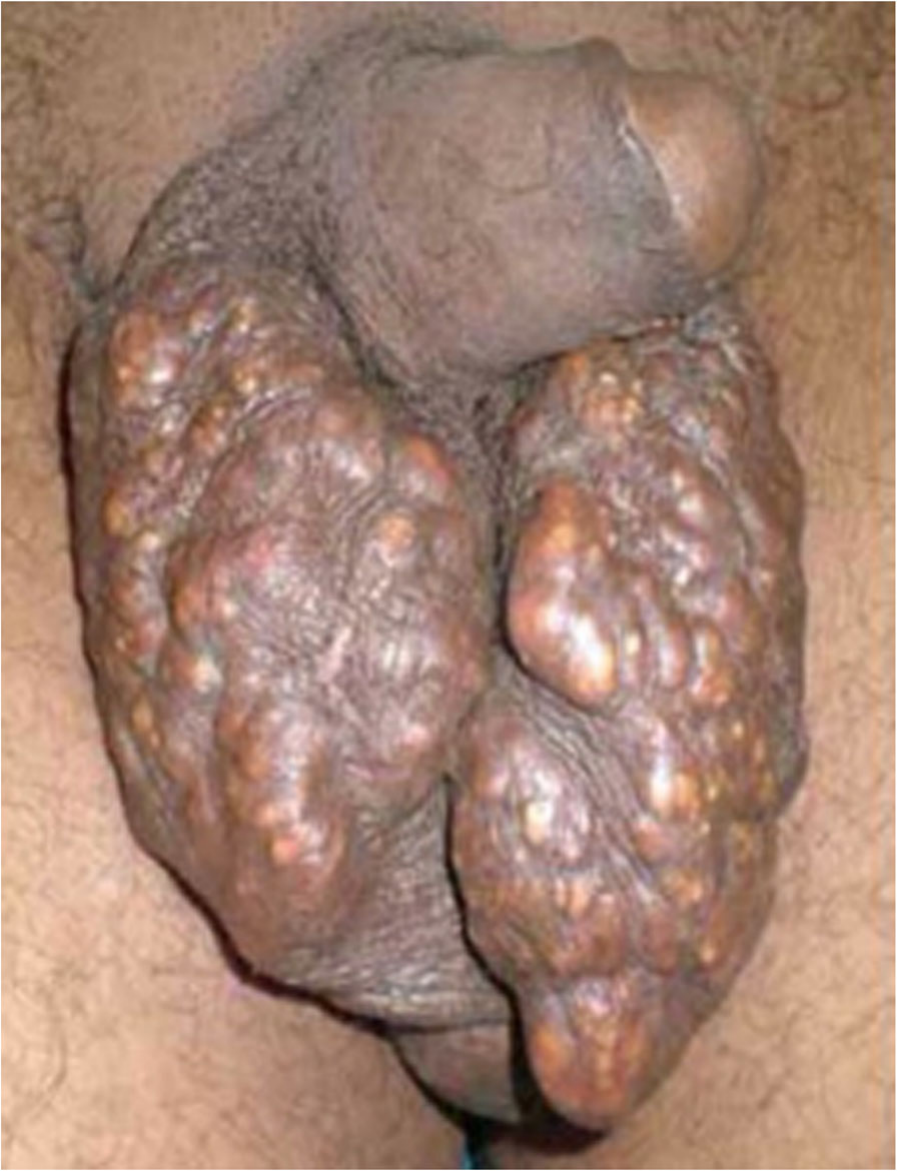
Multiple calcified lesions of the scrotal skin

**Fig. 2:**
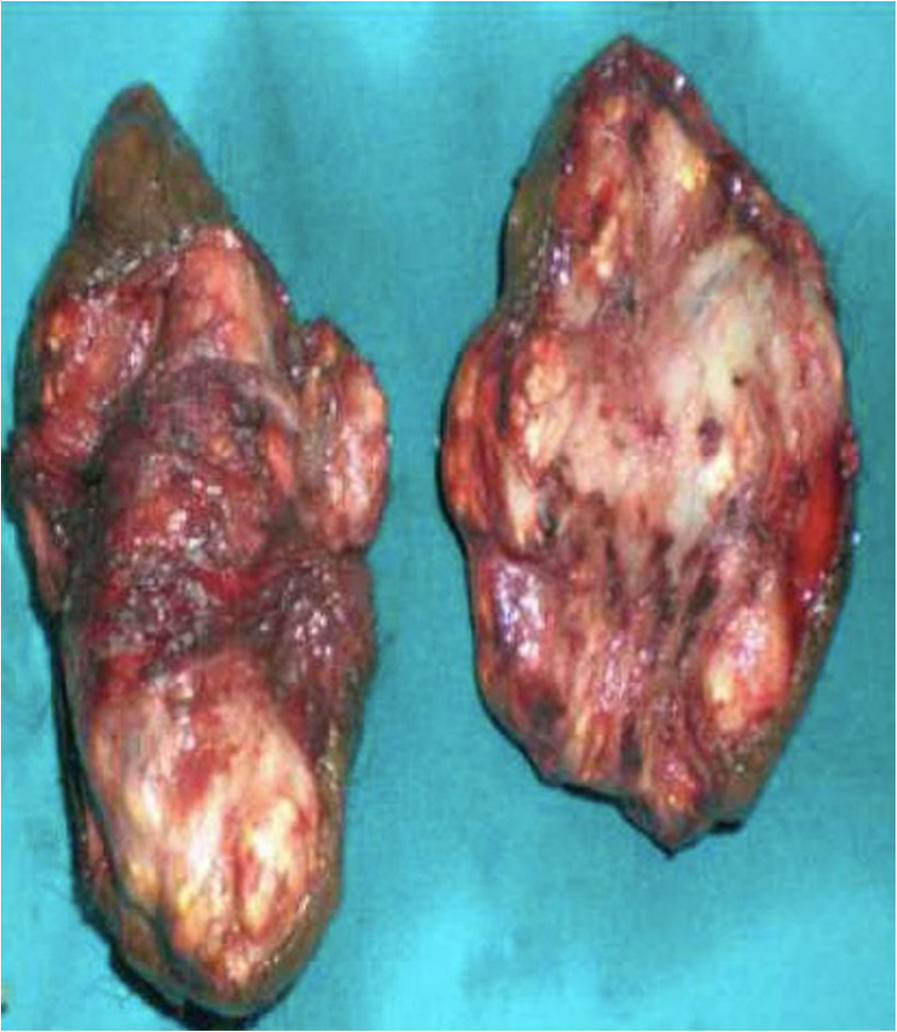
Operative specimen: multiple hard and thick-walled calcified nodules

A 48-year-old North African white married man, a father of two with no past medical history, consulted our institution for calcified nodules of the scrotal skin. He complained of an impact on his sexual performance. The nodules progressively increased in size over 4 months, with a whitish but nonpurulent discharge of the most voluminous nodule. He had no history of traumatism or genitourinary or hormonal disorders. His physical examination revealed multiple and bilateral calcified cutaneous lesions of the scrotum. No inflammatory lesions surrounding the nodules were observed. The nodules ranged in size from 10 to 15 mm (Fig. 1). The described lesions were exclusively scrotal; there were no extensions to the penis. The patient’s blood and urine test results, including mainly calcium, phosphorus, and parathyroid hormone, were normal. He underwent surgery under general anesthesia. A complete resection of the lesions was performed. The operative specimens included hard and thick-walled calcified nodules (Fig. 2). Histological examination revealed a dermal, basophilic, calcified material and an atrophic epithelium. Both nodules were surrounded by an intense fibrosis with multiple giant cells. Immunohistochemical investigation revealed a negative carcinoembryonic antigen, whereas cytokeratin was positive in the surface of epithelial cells. No recurrence has been noted after 5 years of follow-up. The patient was satisfied with the aesthetic result and reported better sexual performance.

### Patient 2

**Fig. 3:**
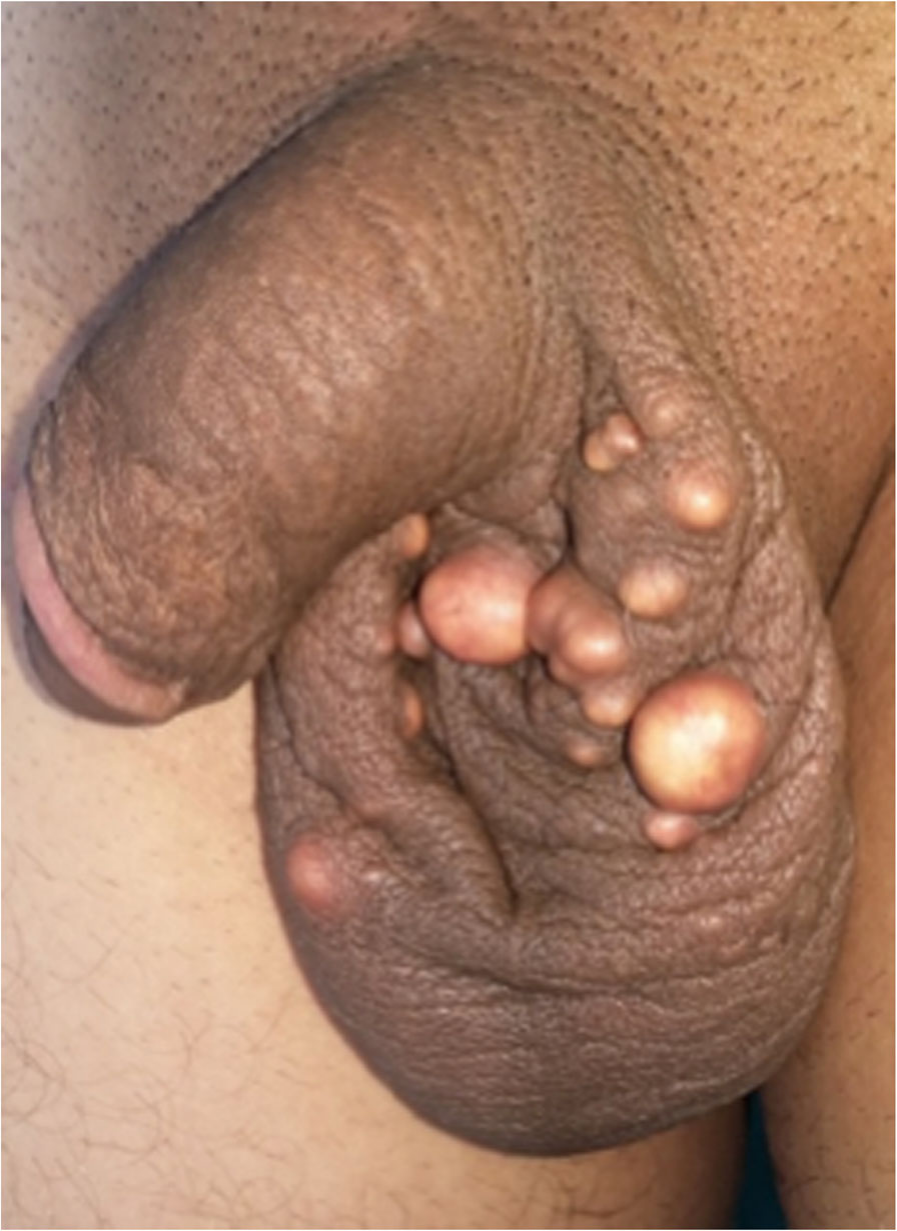
Calcified nodules of the scrotum measuring 5–15 mm

A 28-year-old North African white single man with no particular history presented to our institution with a complaint of a 3-year history of evolving scrotal calcified lesions with a recently increasing volume and subsequent sexual discomfort. The nodules were yellowish, bilateral, and indolent with no discharge or inflammatory signs (Fig. 3). No other locations were noted in the physical examination. Plasma and urine calcium and phosphorus were at normal levels, and the results of routine blood and urine tests were normal.

**Fig. 4:**
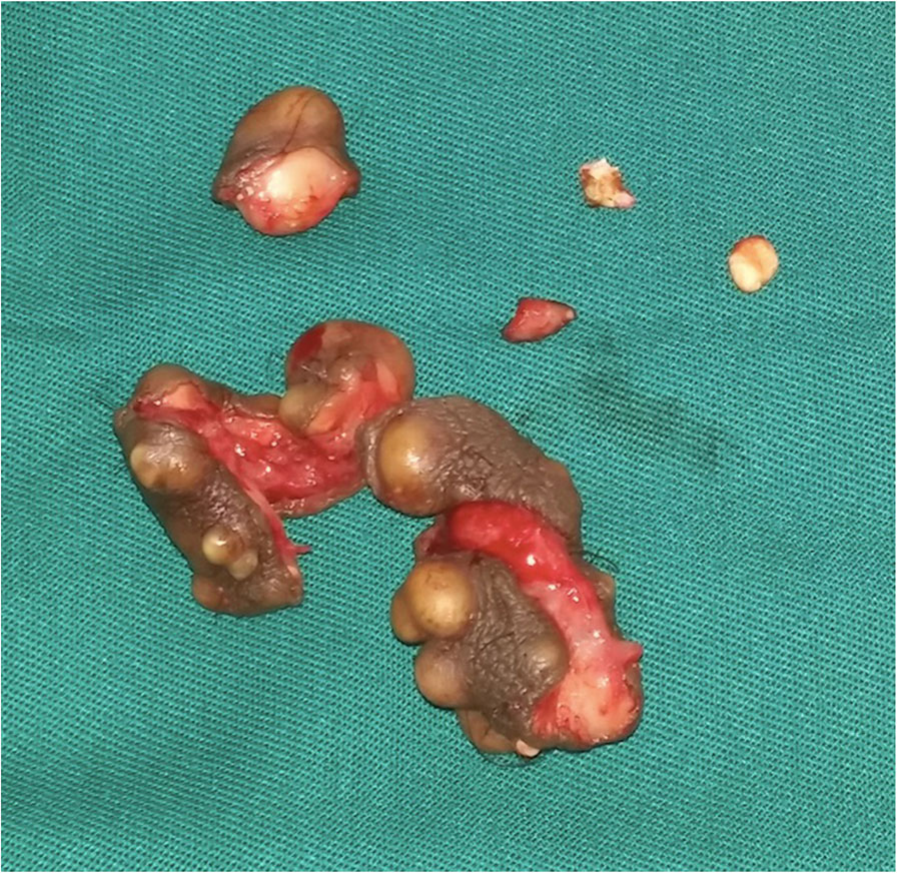
Operative specimen: excision of multiple calcified nodules

Surgery was performed with a fine dissection between the dermis and the dartoic muscle, allowing the excision of ten calcified nodules (Fig. 4). The biggest one measured 20 mm. The patient’s postoperative course was uneventful.

**Fig. 5:**
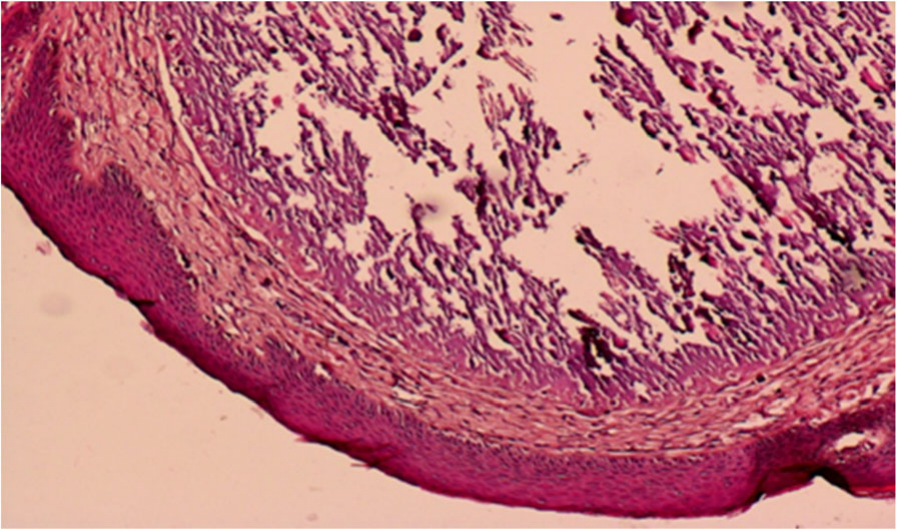
Calcified areas in the dermis

**Fig. 6:**
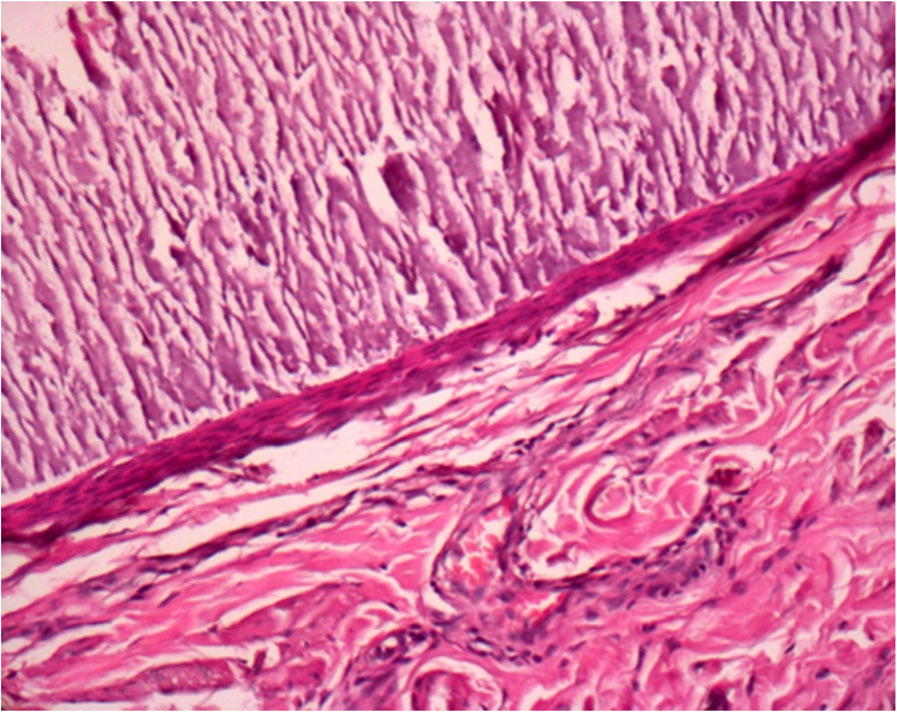
Calcified areas bordered by a Malpighian cell epithelium

Operative specimen and histological examination concerned around ten calcified nodules. The epidermis was regular but thickened and hyperplastic. Confluent calcified areas were observed in the dermis and were surrounded by fibrotic tissue. These calcifications were bordered by Malpighian cell epithelium. There were no signs of active inflammatory or malignant processes (Figs. 5 and 6).

The patient was examined in the outpatient clinic at 3 weeks and 2 months after surgery. The aesthetic result was judged satisfying, with sexual improvement reported by the patient.

## Discussion

Scrotal calcinosis is a benign condition first described by Lewinski over a century ago [1]. It is reported to be more frequent in black patients between the second and fourth decades of life. Some cases have occurred in infants and in elderly patients [2]. A delay between the beginning of symptoms and the first consultation is usually noted because the lesions affect an intimate part of the body.

Lesions are nodular and indolent, without any discharge or inflammatory and bilateral signs. A penile location has been reported for multiple scrotal calcinosis nodules causing sexual discomfort [3]. To our knowledge, only one case of a unilateral form has been published in the English literature [4]. Lesion size ranges from a few millimeters to several centimeters [5].

The question about the etiopathogenesis is not resolved. As scrotal calcinosis is not associated to any metabolic or hormonal disorder, mainly the calcium and the phosphorus metabolism and the parathyroid hormone activity, the idiopathic character was previously approved [6]. Song *et al*., analyzing more than 50 nodules of scrotal calcinosis, concluded that the common characteristic is a calcified dystrophy of epidermal cysts [7]. This theory was widely recognized after the histological and biochemical evaluation of 100 cases of scrotal calcinosis by Dubey *et al*. [8]. Other authors supported the calcified dystrophy of the dartoic muscle [9], but this theory is less convincing than that of Dubey *et al.* Histological examination using Von Kossa staining usually reveals a basophilic, calcified deposit in the scrotal dermis and the calcinosis nodules that is surrounded by giant cell granulomas in an intense foreign body inflammatory reaction [10].

Surgery is usually performed in patients presenting with voluminous lesions carrying a psychological and a sexual prejudice. The total excision of calcified nodules may ignore small lesions, and there is potential for recurrence [5]. Recently, Noel *et al*. [3] proposed a one-stage technique used mainly for giant nodules with a considerable skin defect. Elliptic resection is performed after making an incision centered on the median raphe and a thorough dissection of the scrotal dermis from the dartoic muscle, allowing more pertinent detection of small nodules than the classical resection of nodules detectable on the surface of the skin. This technique is a particularly attractive procedure because the vascularization provided by the external pudendal artery is peripheral [11]. The aesthetic result is better after a median raphe incision and a fine dissection conserving the integrity of the skin integumentary capillaries.

The prognosis after surgery is favorable. Few cases of recurrence have been reported. Recurrence seems to be related to neglected millimetric nodules that increase in size later [5, 12]. No cases of malignant transformation have been reported [8].

## Conclusions

Scrotal calcinosis is a benign condition that occurs in young adults. Clinicians have to reassure patients because the nodules concern an intimate part of the body. Urologists have to keep in mind the aesthetic prejudice and the functional aspect of the surgery. An acceptable restitution of the scrotal skin integument after surgical excision is imperative. Better knowledge of the etiopathogenic aspects of scrotal calcinosis may lead to more efficient surgical procedures with the aim of ensuring better results and preventing recurrences.
